# Gender Discrimination and Excess Female Under-5 Mortality in India: A New Perspective Using Mixed-Sex Twins

**DOI:** 10.1007/s13524-020-00909-0

**Published:** 2020-09-25

**Authors:** Ridhi Kashyap, Julia Behrman

**Affiliations:** 1 Department of Sociology, Nuffield College, and Leverhulme Centre for Demographic Science, University of Oxford, Oxford, UK; 2 Department of Sociology and Institute for Policy Research, Northwestern University, Evanston, IL USA

**Keywords:** Son preference, Under-5 mortality, Excess female child mortality, India, Twins

## Abstract

Son preference has been linked to excess female under-5 mortality in India, and considerable literature has explored whether parents invest more resources in sons relative to daughters—which we refer to as *explicit discrimination*—leading to girls’ poorer health status and, consequently, higher mortality. However, this literature has not adequately controlled for the *implicit* discrimination processes that sort girls into different types of families (e.g., larger) and at earlier parities. To better address the endogeneity associated with implicit discrimination processes, we explore the association between child sex and postneonatal under-5 mortality using a sample of mixed-sex twins from four waves of the Indian National Family Health Survey. Mixed-sex twins provide a natural experiment that exogenously assigns a boy and a girl to families at the same time, thus controlling for selectivity into having an unwanted female child. We document a sizable impact of explicit discrimination on girls’ excess mortality in India, particularly compared with a placebo analysis in sub-Saharan Africa, where girls have a survival advantage. We also show that explicit discrimination weakened for birth cohorts after the mid-1990s, especially in northern India, but further weakening has stalled since the mid-2000s, thus contributing to understandings of how the micro-processes underlying the female mortality disadvantage have changed over time.

**Electronic supplementary material:**

The online version of this article (10.1007/s13524-020-00909-0) contains supplementary material, which is available to authorized users.

## Introduction

Son preference continues to be a defining feature of family life in India, shaping the well-being of Indian women and girls throughout the life course. One of the most striking demographic manifestations of son preference in India is the persistence of excess female infant and child mortality. Despite declining levels of overall under-5 mortality, India continues to experience one of the highest levels of excess female under-5 mortality in the world (Alkema et al. 2014; Guilmoto et al. 2018; Kashyap 2019). The term *excess* implies that girls experience higher than biologically expected levels of mortality relative to boys, which, as famously characterized by Amartya Sen (1990), results in women and girls being “missing” from population structures.^1^

The dominant demographic explanation for the female mortality disadvantage in India has been that parents invest more resources (e.g., immunizations, medical treatment, nutrition) in sons relative to daughters—a set of processes that we refer to as *explicit discrimination*—leading to girls’ poorer health status and, consequently, higher mortality (Caldwell and Caldwell 1990; Caldwell et al. 1982; Das Gupta 1987; Miller 1981). However, research linking explicit discrimination to excess female child mortality in India confronts a major measurement issue: the sex^2^ composition of families is not random in India, and the literature typically does not adequately control for the passive son-preferring fertility behaviors—which we call *implicit discrimination*—that sort girls into different types of families and at earlier parities within families. For example, son-preferring fertility-stopping rules imply that families may continue to have children until their desired number of sons is reached, which results in girls being born into larger families (Clark 2000; Filmer et al. 2009; Rosenblum 2013; Yamaguchi 1989) and at earlier parities relative to boys (Basu and De Jong 2010). Furthermore, prenatal sex selection in the form of sex-selective abortion allows some families to opt out of having daughters (Anukriti et al. 2018; Bhalotra and Cochrane 2010; Hu and Schlosser 2015; Jha et al. 2006; Kashyap 2019). If technology access to practice sex selection is concentrated in urban, better-off families, then girls may be disproportionately sorted into disadvantaged households. The conceptual implication of different implicit discrimination processes is that the population-level female mortality disadvantage could emerge from girls being disproportionately born into larger and/or poorer families where overall mortality is higher (i.e., implicit discrimination) rather than differential resource allocation within the same family (i.e., explicit discrimination).

It is empirically difficult to measure explicit discrimination net of implicit discrimination because prenatal sex selection remains unobserved at the family level and because other forms of differential selection, such as family size or birth order, are endogenous to mortality and parental son preference. It is further complicated to explore how explicit discrimination has changed over time given that the implicit processes that might sort girls into qualitatively different households have also changed with diffusion of ultrasound technology, fertility declines, improvements in women’s education, and other social and economic changes. Nonetheless, it is fundamental for our understanding of son preference in India to understand whether explicit discrimination underpins observed mortality disadvantage for girls. Furthermore, accurately documenting explicit discrimination is essential from a policy perspective because the policy responses implied by parents’ differential resource allocation to boys versus girls within families are different than if girls’ mortality disadvantage accrues primarily from selection into different families. If there is intrahousehold discrimination against girls, then policies targeting health and welfare investments in girls specifically within families will be necessary. However, if girls fare worse, on average, because of selection into larger and/or poorer families, then policies addressing generalized poverty reduction will be better motivated.

To better address the endogeneity of implicit discrimination processes, we use a large sample of mixed-sex twins to investigate the association between child sex and postneonatal under-5 mortality with data from four waves of the Indian National Family Health Survey (1992–1993, 1998–1999, 2005–2006, 2015–2016). Mixed-sex twins provide a natural experiment that exogenously assigns both a boy and a girl to families at the same time, thus allowing us to control for differential family selectivity into having an unwanted female child and other implicit discrimination processes. To validate our estimates of explicit discrimination, we conduct a placebo analysis using a large sample of twins from sub-Saharan Africa, a region that does not have a history of female mortality disadvantage. We also explore heterogeneity in explicit discrimination by stratifying by region and number of older female siblings. Finally, we investigate whether explicit discrimination has declined over subsequent birth cohorts, thus providing important insight into how the micro-processes that contribute to the female mortality disadvantage have changed over time in contemporary India.

## Explicit and Implicit Discrimination Processes That Contribute to the Female Under-5 Mortality Disadvantage in India

Patterns of excess female infant and child mortality arising from a strong preference for male offspring have been long noted in India. Sex ratios (male/female) of infant and child mortality in India have remained under 1.00, which is indicative of a female mortality disadvantage that is attributable to social (gender) discrimination processes because males are biologically more vulnerable to mortality in infancy and, to a lesser extent, in early childhood. Particularly in the age group 1–4 years, India has the most anomalous levels of excess female mortality in the world (Alkema et al. 2014; Guilmoto et al. 2018; Kashyap 2019). In what follows, we highlight both explicit and implicit discrimination processes that contribute to the female under-5 mortality disadvantage, with discussion of how these processes may have changed over time.

Excess female infant and child mortality has long been thought to arise from explicit postnatal discrimination against girls in the allocation of resources, such as health care (e.g., immunizations, medical treatment) or nutrition (e.g., food, breastfeeding) that are relevant for survival (Arnold et al. 1998, Das Gupta 1987; Miller 1981; Mishra et al. 2004; Pande 2003). Nonetheless, the empirical evidence for sex-differential allocation of resources has often been mixed or inconclusive. Whereas several studies have found that girls were less likely to receive health care and vaccinations (Borooah 2004; Corsi et al. 2009; Ganatra and Hirve 1994; Mishra et al. 2004; Pande 2003; Rajan and Morgan 2018), others have found similar vaccination rates for boys and girls (Barcellos et al. 2014; Deaton 2003). Evidence on sex differences in children’s diet is similarly mixed (Basu 1989; Fledderjohann et al. 2014). Population-level studies of anthropometric measures, such as malnutrition and stunting, also show an ambiguous picture with no clear female disadvantage in these measures and in some cases show a male disadvantage in the first 24 months of life albeit with the gap closing at later ages (Corsi et al. 2015; Mishra et al. 2004). Studies, however, have found a clear female disadvantage with respect to duration of breastfeeding (Barcellos et al. 2014; Fledderjohann et al. 2014; Jayachandran and Kuziemko 2011). In some cases, though, son preference may actually disfavor boys, who may be exclusively breastfed longer in the ages between 6–9 months when breastfeeding alone is not sufficient to meet an infant’s energy needs (Mishra et al. 2004). Parents in India have also been shown to make differential prenatal investments in male versus female fetuses (Bharadwaj and Lakdawala 2013).

In seeking to reconcile these mixed findings, which on one hand show a female disadvantage in mortality linked to son preference but weaker evidence for sex differences in resources and anthropometric measures, studies have argued that the female disadvantage is not generalized but rather concentrated among certain subsets of girls, particularly those born at later parities and without brothers. These birth order and sibling composition effects have been found both in terms of health inputs and outcomes (Mishra et al. 2004; Pande 2003) as well as in mortality (Arnold et al. 1998; Muhuri and Preston 1991). According to this perspective, son preference does not imply that all girls are unwanted, but daughters deemed to be “redundant” are more likely to experience discrimination. In contrast to these perspectives, Rajan and Morgan (2018) argued that generalized discrimination against girls—affecting all daughters at a given parity rather only than those with older sisters and no brothers—provides a better description of patterns of female disadvantage observed in India for outcomes such as immunization, treatment for respiratory illness, and breastfeeding after 17 months.

Most of the aforementioned studies have estimated the effect of being female on a mortality outcome or particular investment (e.g., immunization, breastfeeding) and compared boys and girls between different families, usually in a regression framework. However, in a context where son preference shapes fertility behaviors and family sex composition is not random, girls are likely to be selected into different types of families. This differential selection—which we term *implicit discrimination*—may also affect the observed aggregate-level disadvantage in girls’ outcomes. As we describe later, controlling for different forms of implicit discrimination, which may be changing over time, is important for detecting whether there is gender discrimination within families that leads to excess female mortality, but controlling for this is not always straightforward.

For example, studies have found that son-preferring fertility rules result in girls being born into larger families—that is, having larger siblingship sizes relative to boys (Basu and De Jong 2010; Clark 2000; Rosenblum 2013; Yamaguchi 1989). As a result, if fewer resources per family member are available in larger families, girls will, on average, share resources with larger sibling cohorts and be worse off when compared with boys. Conversely, if there are returns to scale for certain resources in large families, then girls might not be worse off but could benefit from these instead (Barcellos et al. 2014). Rosenblum (2013) found these son-preferring stopping rules that result in girls being born into larger families exacerbate excess female child mortality in India. Couples with firstborn boys had fewer total children born and a higher proportion of males in their families. Using reduced form estimates, Rosenblum found that second- and higher-order girls in families with firstborn sons had 25% higher mortality than boys, whereas those in households with firstborn girls had 38% higher mortality than boys.

Another implication of son-preferring fertility behavior is that girls are more likely to be born at earlier parities within families (Basu and De Jong 2010). The implication of this form of selection for mortality is *a priori* ambiguous. Whereas some studies found a J-shaped relationship between infant mortality and birth order (e.g., Hobcraft et al. 1985; Pandey et al. 1998), with firstborns showing the elevated risks, others found a linearly increasing risk from earlier-born to later-born children, which could protect earlier-born girls (e.g., Mishra et al. 2018). Basu and De Jong (2010) hypothesized that in a context with son preference, earlier-born daughters may experience negative consequences for their well-being as a result of having to assist in the care of later-born children within their families.

Yet another form of selection that may affect the family conditions into which girls are born is prenatal sex selection, most commonly in the form of sex-selective abortion. The practice of prenatal sex selection began in the early 1990s, as indicated by distorted sex ratios at birth, especially in northern Indian states of Punjab, Haryana, Delhi, and others (Guilmoto 2015; Hu and Schlosser 2015; Jha et al. 2006). Whether prenatal sex selection protects or worsens girls’ mortality outcomes depends on which families are able to access it. If sex-selective abortion enables households with the strongest son preference, and thus those that might have otherwise resorted to explicit discrimination, to avoid having unwanted daughter(s) and reduce family size, this form of implicit discrimination may protect girls (Goodkind 1996; Hu and Schlosser 2015; Kashyap 2019). However, uneven access to technology enabling sex selection may imply that wealthier families are better able to avoid unwanted female births, and girls may be differentially sorted into households with overall fewer resources because these households cannot afford to opt out of having daughters even if sons are preferred (Hu and Schlosser 2015; Kashyap 2019). This may worsen girls’ population-level disadvantage. Studies from India have found that sex ratios at birth are most distorted among wealthier, urban, and more educated families (Jha et al. 2006), which has generally been interpreted as a sign of better access to ultrasound technology among these groups (Guilmoto 2015).

Aggregate indicators—such as the sex-disaggregated under-5 mortality rate—mask both explicit and implicit discrimination processes and cannot adequately capture whether the micro-level processes of explicit discrimination have changed over time. Explicit discrimination could have changed in India through different channels. Some scholars have suggested that diffusion of ultrasound technology could lead to a substitution whereby postnatal discrimination in the allocation of nutrition and health resources between male and female children (e.g., explicit discrimination) is weakened as a result of the uptake of prenatal discrimination via sex-selective abortion (e.g., implicit discrimination) (Goodkind 1996; Kashyap 2019; Sen 2003). Evidence for this hypothesis in the Indian context has so far been mixed. Whereas Hu and Schlosser (2015) did not find faster reductions in girls’ mortality relative to boys for cohorts that witnessed prenatal sex selection, Anukriti et al. (2018) reported evidence for faster reductions in girls’ mortality in the period when ultrasound became widely available in India (after 1995). Disentangling the effects of weakening son preference from the practice of sex selection is empirically challenging, however, and son preference can be weakening even as sex ratios at birth become more masculine (Kashyap and Villavicencio 2016). Sex-selective abortion may enable families to reconcile son preference with a small family size and thus also facilitate fertility decline in contexts with son preference (Jayachandran 2017; Kashyap and Villavicencio 2016). On the other hand, explicit discrimination could also decline as a result of weakening son preference. Son preference, as measured by different indicators of ideal sex composition, may be weakening in India with wider processes of socioeconomic development and fertility decline (Bhat and Zavier 2003; Bongaarts 2013; Kashyap and Villavicencio 2017; Retherford and Roy 2003).

### Accounting for Implicit Discrimination in Measurement of Explicit Discrimination

A standard approach for measuring the mortality attributable to explicit discrimination would be to estimate the association between child sex and mortality controlling for birth order, family size, family socioeconomic status (SES), and other measures related to the implicit discrimination processes that sort girls and boys into different families. However, with this approach, it would be difficult to appropriately control for implicit discrimination processes because the intensity of son preference and prenatal sex selection are unobserved at the family level, and variables such as completed family size and birth order are endogenous to mortality and parental son preference. Controlling for variables such as family size or sex composition would allow us to compare outcomes of boys and girls in families of the same size. However, as Barcellos et al. (2014) noted, in the presence of son-preferring stopping rules, if we compare girls and boys in families of the same size, girls are, on average, in families that desire fewer sons (than the family of the average child). In other words, even conditional on family size and sex composition, child sex is not exogenous but is correlated with parental preferences for the sex composition of children.

One approach for addressing the endogeneity of family size, as followed by Rosenblum (2013), has been to capture exogenous variation related to son-preferring stopping rules by using the sex of the first child as a natural experiment.^3^ Although this approach demonstrates how sex-differential stopping rules exacerbate mortality outcomes, it cannot estimate an effect of explicit discrimination, net of implicit discrimination, for girls. An alternative approach, used by Barcellos et al. (2014) to examine whether boys and girls receive differential resources, has been to focus on the youngest child in the family—when they are young enough, and the next birth has not yet occurred—to measure boy-girl differences in this sample. In the absence of sex-selective abortion, the sex of the child in this sample can be assumed to be exogenous; consequently, their study focused on births that occurred before the 1990s, after which sex-selective abortion became practiced in India. Both strategies, by Rosenblum (2013) and Barcellos et al. (2014), are less effective in a context where sex-selective abortion is practiced and cannot adequately address changes in explicit discrimination over time.

We propose a novel strategy to better address the endogeneity associated with implicit discrimination processes by leveraging mixed-sex twins as a natural experiment in which both a boy and a girl are assigned to a family at the same time, thus allowing us to control for implicit discrimination processes, such as differential family selectivity into having an unwanted female child. Mixed-sex twins, exposed to the same prenatal environment and born at the same time, are exposed to the same family environment (e.g., wealth at birth). Because the principal difference in mixed-sex twins is child sex—and not family size, birth order, maternal age, family wealth at birth, and so on—elevated female mortality among mixed-sex twins should be more readily attributable to differential parental behaviors based on child sex (e.g., explicit discrimination). This is particularly the case given that biologically male children are more vulnerable to death in infancy and early childhood (Drevenstedt et al. 2008), and this has also been shown to hold for twin populations (Ahrenfeldt et al. 2017; Pongou 2013). Thus, in a context without differential treatment of male and female children, we would actually expect a female survival advantage.

## Data and Sample

We use pooled standardized data on mixed-sex twins from the 1992–1993, 1998–1999, 2005–2006, and 2015–2016 India National Family Health Survey (NFHS). The NFHS collects cross-sectional microdata on key demographic and health outcomes that are nationally representative of all women ages 15–49 and follows the format and structure of the Demographic and Health Surveys (DHS). The NFHS is collected by the Indian Ministry of Health and Family Welfare and International Institute for Population Sciences, with input from ORC Macro International.

We identify a sample of mixed-sex twins using the NFHS birth recode, which provides detailed information on all children born to women in the sample. Respondents are queried about whether each child is still alive and, if not, at what age in months the child died. For each birth, respondents are also asked whether it is a multiple birth (e.g., twin birth), the birth order, and the sex. Combining this information allows us to identify which births are mixed-sex twins. We exclude households with multiple sets of twins and households with triplets and quadruplets given the exceptional nature of these events, which suggests that these household might be categorically different from others in the sample in their potential genetic predisposition to twinning. Our analytical sample includes 6,200 mixed-sex twins from 3,100 families.

## Empirical Approach

### Measures

#### *Mortality:*

Our main outcome is a dichotomous indicator of whether the birth resulted in death in infancy or early childhood (e.g., between 1 and 59 months). We exclude mortality in the first month of life because we are interested in capturing social rather than biological processes that impact mortality, and previous literature on son preference has shown that its mortality manifestations in under-5 mortality are most apparent in the postneonatal ages (Das Arnold et al. 1998; Arokiasamy 2004; Das Gupta 1987). All infant and child deaths are self-reported by mothers and thus are subject to reporting bias. Nonetheless, because the death of an offspring is a rare and important event, it is reasonable to believe that mothers would accurately remember the age of offspring death.

#### *Child sex:*

Throughout the models, the main treatment outcome is a dichotomous indicator of whether the birth was female.

#### *Firstborn twin:*

We include a control for which twin was born first because on average, firstborn twins are heavier than second-born twins, which may have implications for parental investment and later-life outcomes (Pongou 2013).

### Estimation Strategy

We use a within-twin fixed-effects model that allows us to compare boy-girl differences in mortality within twin pairs born into the same family. The fixed effect (in Eq. (1), α_*i*_) captures all observed and unobserved factors (e.g., family SES and environment, prenatal inputs) shared between the twin pair. For an individual *j* in twin pair *i,* the within-twin fixed-effects model of sex of the child on mortality can be expressed as follows:

1 $$ Mort\mathrm{a}{lity}_{ij}={\upbeta}_0+{\upbeta}_1{Firstborn}_j+\uptau {Female}_j+{\upalpha}_i+{\upvarepsilon}_{ij}. $$

First, we use the within-twin fixed-effects model in Eq. (1) to estimate the effect of being female (τ) on the probability of postneonatal under-5 mortality pooling across the four NFHS survey waves. We interpret τ as a measure of the female mortality disadvantage that is attributable to explicit discrimination. We use linear probability models throughout analyses for the ease of interpretation and comparability of coefficients across models. Furthermore, we assess whether there is evidence of changes in explicit discrimination by running models across three birth cohorts of mixed-sex twins: (1) twins born prior to 1995, (2) twins born between 1995 and 2005, (3) and twins born after 2005. Our birth cohorts roughly correspond with those suggested by Anukriti et al. (2018) regarding different periods of diffusion of ultrasound technology in India, whereby the first period represents the early diffusion period when ultrasound was still new and less common, the second period represents a period by which ultrasound use is widespread, and the third period corresponds with more recent history in India.^4^ After the mid-1990s, implicit discrimination associated with sex-selective abortion—in addition to sex-differential stopping behavior—became possible because of the diffusion of ultrasound technology. The measurement problems introduced by sex-selective abortion do not affect the within mixed-sex twin estimates because sex-selective abortion is not a viable option for mixed-sex twins. Nevertheless, this form of implicit discrimination shapes the broader context of the families into which mixed-sex twins get randomly assigned over this period: for example, after the mid-1990s, parents could use sex-selective abortion to implement their son preference prior to having twins or for future children.

To validate our measure, we also conduct a placebo analysis using a large sample of sub-Saharan African (SSA) twins. The SSA data come from the Demographic and Health Surveys (standardized with the NFHS), with multiple survey waves occurring over the same period as the NFHS (see Table A1 in the online appendix for further detail on the SSA sample). Because SSA does not show patterns of son-preferring fertility behaviors (Basu and De Jong 2010; Jayachandran and Pande 2017) and aggregate-level under-5 female mortality disadvantage (Alkema et al. 2014), we hypothesize that the females in mixed-sex twin pairs in this region should not experience a mortality disadvantage. Thus, if there is a female mortality disadvantage among mixed-sex twins in India but not SSA (i.e., a higher magnitude of τ in India compared with SSA), this is further evidence of social—as opposed to biological—processes leading to the under-5 mortality disadvantage.

Given evidence that suggests heterogeneity in son preference in India—including by region and by family structure—we also rerun the within-twin fixed-effects models stratifying by region and number of older sisters. Regional variations in son preference and its manifestations have long been noted in India, with son preference notably stronger in northern states (Arnold et al. 1998; Arokiasamy 2004; Bhat and Zavier 2003; Dyson and Moore 1983).^5^ As a further extension, we also stratify by both region and birth cohort to see whether there are changes over birth cohorts at the regional level. Finally, we rerun our mixed-sex twin fixed-effects models stratifying by number of older sisters because explicit discrimination may be more common in families where there are multiple older sisters (Arnold et al. 1998; Mishra et al. 2004; Pande 2003).

## Results

### Descriptive Summary

Table 1 presents weighted proportions (and means for continuous variables) for descriptive characteristics of our twin sample, including how twin characteristics changed over subsequent birth cohorts. On average, about 9% of the twins in our pooled sample died before age 5, although survival improved over time. For example, 17% of twins born before 1995 died before age 5, compared with 7% and 5% of twins born between 1995 and 2005 and after 2005, respectively.

**Table 1:** Descriptive summary of background characteristics of mixed-sex twins, including tests for significant difference between the first-birth cohort and the two subsequent cohorts

	Pooled	Born Before 1995	Born in 1995–2005	Born After 2005
	(1)	(2)	(3)	(4)
Mortality (1–59 months)	0.09	0.17	**0.07**	**0.05**
Female	0.50	0.50	0.50	0.50
Birth Year	2000	1986	**2000**	**2011**
Birth Order	3.26	3.68	**3.18**	**2.98**
Rural	0.68	0.69	0.66	0.68
Northern Region	0.60	0.61	0.58	0.60
Hindu	0.79	0.77	0.80	0.79
Poorest 40%	0.40	0.36	0.38	**0.46**
Mother No School	0.45	0.58	**0.44**	**0.33**
Mother Primary School	0.16	0.20	0.17	**0.12**
Mother Secondary School	0.30	0.19	**0.32**	**0.39**
Mother Tertiary	0.09	0.04	**0.07**	**0.16**
Mother Age at Birth	25.32	24.68	24.92	**26.34**
Total Children Born to Mother	4.32	5.22	**4.19**	**3.69**
Mother’s Ideal Number of Boys	1.36	1.56	**1.31**	**1.26**
Mother’s Ideal Number of Girls	1.08	1.14	**1.07**	**1.05**
Mother’s Ideal Sex Ratio	0.55	0.57	**0.55**	**0.54**
*N*	6,200	1,868	2,278	2,054

*Notes*: All estimates use pooled data from the Indian National Family Health Survey (1992–1993, 1998–1999, 2005–2006, 2015–2016) and use sampling weights provided by the NFHS. All measures are dichotomous except birth year (ranges from 1958 to 2016), birth order (ranges from 1 to 11), mother age at birth (ranges from 12 to 47), total number of children born (ranges from 2 to 13), mother’s ideal number of boys (ranges from 0 to 7), mother’s ideal number of girls (ranges from 0 to 6), and ideal sex ratio (ranges from 0 to 1 and excludes women who desire 0 children). Bold numbers indicate a statistically significant (*p* < .05) difference between the birth cohort in question and the first birth cohort (i.e., born before 1995). Two-sample *t* tests are performed for all continuous outcomes, and chi-square tests are performed for all dichotomous outcomes.

These declines in mortality likely correspond with a number of other important changes in fertility and family life that also occurred over subsequent birth cohorts. For example, between the oldest (e.g., born before 1995) and youngest (e.g., born after 2005) birth cohorts, twins were increasingly born into smaller families at earlier birth orders, which corresponds with overall fertility declines in India in recent history. Mothers in the sample are increasingly better educated and have children at older ages, which also makes sense given that these are key correlates of fertility declines. Mother’s stated ideal number of boys and girls also both significantly declined over subsequent birth cohorts of twins, which may reflect a preference for increasingly smaller family sizes. There are also significant declines over birth cohorts in mother’s ideal sex ratio of boys to girls (ideal boys divided by ideal number of children), which does suggest some lessening of stated son preference over time. Nonetheless, even in the most recent birth cohort, mother’s ideal sex ratio is still skewed toward boys (0.54).

### Exploring Explicit Discrimination and the Female Postneonatal Under-5 Mortality Disadvantage Using Within-Twin Fixed Effects

To better estimate the effects of explicit discrimination, we start by using a pooled sample of mixed-sex twins to conduct a within-twin fixed-effects analysis of the association between child sex and postneonatal under-5 (1–59 months) mortality. Unlike past estimates of the female mortality disadvantage, this specification allows us to control for implicit discrimination processes by accounting for unobserved twin-level confounders that do not vary between twins (e.g., prenatal conditions, family SES, family size, birth timing, and birth order). We find that females experience a 2 percentage point higher probability of postneonatal under-5 mortality than males (*p* < .01) (Table 2, panel A, column 1), which corresponds to a 27% higher mortality for girls relative to the mortality probability for boys of the pooled sample (.075). These results provide strong evidence of explicit discrimination playing an important role in the female mortality disadvantage observed in our data, net of implicit discrimination processes. The substantive findings from Table 2 are robust to respecification as logistic regression fixed-effects and Cox proportional hazard models in which the outcome is age (in months) at death (online appendix, Table A2, panels A and B). Results are also robust to limiting to children born within 10 years of the survey to minimize recall bias in the reporting of children’s age at death (Table A2, panel C).

**Table 2:** Within mixed-sex twin fixed-effects models of the association between child sex and infant and child mortality (1–59 months) in India (panel A) and sub-Saharan Africa (SSA) (panel B)

	Mortality 1–59 Months, Pooled	Mortality 1–59 Months, Born Before 1995	Mortality 1–59 Months, Born in 1995–2005	Mortality 1–59 Months, Born After 2005
	(1)	(2)	(3)	(4)
A. India				
Female	0.020**	0.053***	**0.006**	**0.007**
	(0.006)	(0.015)	**(0.010)**	**(0.009)**
Firstborn twin	–0.039***	–0.023	–0.047***	–0.042***
	(0.006)	(0.015)	(0.010)	(0.009)
Constant	0.097***	0.131***	0.093***	0.069***
	(0.006)	(0.013)	(0.009)	(0.008)
Number of observations	6,200	1,868	2,278	2,054
* R*^2^	.017	.019	.021	.023
Number of families	3,100	934	1,139	1,027
Baseline male mortality	0.075	0.118	0.068	0.046
B. Sub-Saharan Africa (SSA)				
Female	–0.016***	–0.024**	–0.008	–0.013
	(0.005)	(0.008)	(0.007)	(0.009)
Firstborn twin	–0.042***	–0.066***	–0.037***	0.001
	(0.005)	(0.008)	(0.007)	(0.009)
Constant	0.209***	0.280***	0.189***	0.098***
	(0.004)	(0.007)	(0.007)	(0.008)
Number of observations	17,963	7,124	7,533	3,306
* R*^2^	.009	.018	.007	.001
Number of families	8,988	3,566	3,767	1,655
Baseline male mortality	0.185	0.243	0.169	0.099

*Notes:* Estimates use pooled data from the Indian National Family Health Survey (1992–1993, 1998–1999, 2005–2006, 2015–2016) and the Demographic and Health Surveys in Africa. See Table A1 in the online appendix for full list of SSA countries and survey waves. Analysis were conducted in STATA 15. Bold numbers indicate a statistically significant (*p* < .05) difference between the birth cohort in question and the first birth cohort (i.e., born before 1995).

**p < .01; ***p < .001

Over the time span covered in our study, son preference and forms of implicit discrimination changed with fertility declines, socioeconomic development, and diffusion of ultrasound technology to facilitate sex-selective abortion. Our next step is to explore how mortality attributable to explicit discrimination changed over subsequent birth cohorts. As panel B of Table 2 shows, among mixed-sex twins born before 1995 (e.g., before the widespread diffusion of ultrasound technology and uptake of prenatal sex selection), females experience a 5.3 percentage point higher probability of postneonatal under-5 mortality compared with males (*p* < .001) (Table 2, panel A, column 2), or a 45% higher mortality relative to male mortality of this period. On the other hand, females experience 0.6 percentage points (or 9%) and 0.7 percentage points (or 15%) higher postneonatal under-5 mortality compared with males for the latter two cohorts, those born in 1995–2005 and after 2005 (Table 2, panel A, columns 3–4), respectively (neither coefficient is statistically significantly different from 0 at *p* < .05). The female coefficient in the earliest birth cohort (e.g., before 1995) is significantly higher than the female coefficients in the latter two birth cohorts, thus providing evidence of the female mortality disadvantage attributable to explicit discrimination weakening between the first cohort and the two subsequent ones. The coefficient between the second and third cohort are similar in magnitude and indicate a stagnation of improvements after the mid-2000s. Respecifying the birth cohorts by decades (e.g., born in or before the 1980s; born in the 1990s; born in the 2000s in panel D of Table A2, online appendix) yields substantively similar results, with a significantly elevated female mortality before the 1990s, both in absolute and relative measures, compared with the two successive cohorts. The changes that we document over subsequent birth cohorts are important given the difficulties of empirically assessing whether the micro-level processes that underlie the female under-5 mortality disadvantage have changed over time in the context of changing forms of implicit discrimination.

**Table 3:** Within mixed-sex twin fixed-effects models of the association between child sex and infant and child mortality (1–59 months) for northern regions (panel A) and other regions (panel B)

	Mortality 1–59 Months, Pooled	Mortality 1–59 Months Born Before 1995	Mortality 1–59 Months Born in 1995–2005	Mortality 1–59 Months Born After 2005
	(1)	(2)	(3)	(4)
A. Northern Regions				
Female	0.036***	0.091***	**0.006**	**0.019**
	(0.009)	(0.020)	**(0.014)**	**(0.013)**
Firstborn twin	–0.047***	–0.013	–0.060***	–0.063***
	(0.009)	(0.020)	(0.014)	(0.013)
Constant	0.108***	0.127***	0.113***	0.082***
	(0.008)	(0.018)	(0.013)	(0.012)
Number of observations	3,482	1,074	1,248	1,160
*R*^2^	.028	.042	.030	.047
Number of families	1,741	537	624	580
Baseline male mortality	0.080	0.119	0.079	0.047
B. Other Regions				
Female	–0.001	0.004	0.003	–0.010
	(0.009)	(0.021)	(0.013)	(0.011)
Firstborn twin	–0.026**	–0.032	–0.031*	–0.014
	(0.009)	(0.021)	(0.013)	(0.011)
Constant	0.083***	0.134***	0.070***	0.052***
	(0.008)	(0.019)	(0.011)	(0.010)
Number of observations	2,718	794	1,030	894
*R*^2^	.006	.006	.011	.005
Number of families	1,359	397	515	447
Baseline male mortality	0.069	0.116	0.054	0.045

*Notes:* All estimates use pooled data from the Indian National Family Health Survey (1992–1993, 1998–1999, 2005–2006, 2015–2016). Analysis conducted in STATA 15. Bold numbers indicate a statistically significant (*p* < .05) difference between the birth cohort in question and the first birth cohort (i.e., born before 1995).

*p < .05; **p < .01; ***p < .001

#### *Within Mixed-Sex Twin Placebo Analysis Using Data From SSA Twins*

To validate our within-twin measure, we conduct a placebo analysis using data on mixed-sex twins from SSA (for an overview of the SSA sample, see panel A of Table A3, online appendix). In the SSA analysis, we find that females experience a 1.6 percentage point lower probability of infant and child mortality compared with males (*p* < .001) (Table 2, panel B, column 1), which corresponds to an 8.6% female advantage relative to male mortality in the sample. This finding is consistent with literature suggesting that males are biologically more vulnerable to infant and child mortality than females, which means that in a context without sex differences in allocation of resources, there should actually be a female under-5 mortality advantage (Drevenstedt et al. 2008; Pongou 2013). Upon disaggregating by birth cohort, we find no evidence of statistically significant differences in the female coefficient across the three birth cohorts among mixed-sex twins in SSA (Table 2, panel B, columns 2-4), unlike patterns of change in India. The fact that the SSA results are very different than those presented in India provides further support that the elevated female mortality observed in our India twin sample captures explicit discrimination behaviors toward daughters. The India and SSA comparative results are robust to a pooled difference-in-difference analysis (panel B of Table A3, online appendix). The female × India interaction in panel B of Table A3 indicates that female mortality is about 3.5 percentage points higher in India relative to SSA, which corresponds to a 22% relative excess female mortality compared with male mortality in the pooled sample.

### Exploring Explicit Discrimination in Infant and Child Mortality Separately Using Within-Twin Fixed Effects

**Fig. 1:**
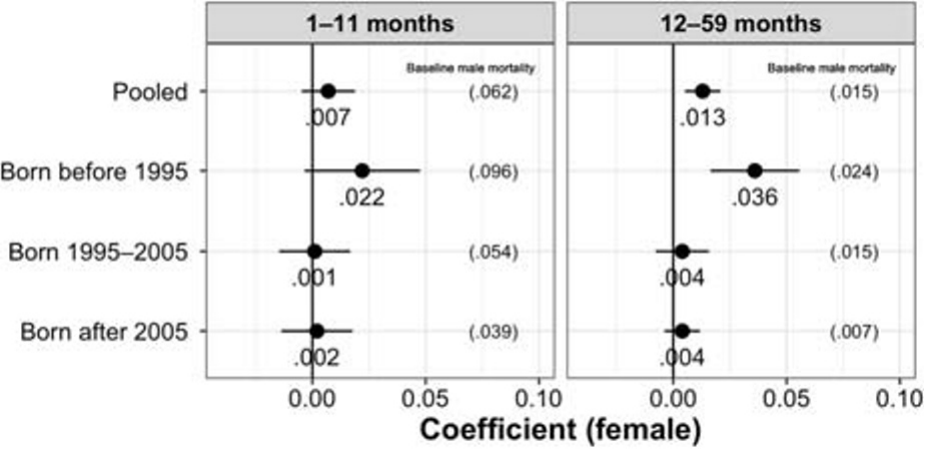
Results of within mixed-sex twin fixed-effects analyses of the effect of child sex on infant mortality (1–11 months) (left panel) and within mixed-sex twin fixed-effects analyses of the effect of child sex on child mortality (12–59 months) (right panel). The baseline male mortality probability of the relevant sample is shown in parentheses. The bars around the point estimate correspond to the 95% confidence interval. *Source:* Created by the authors using data from the NFHS.

**Fig. 2:**
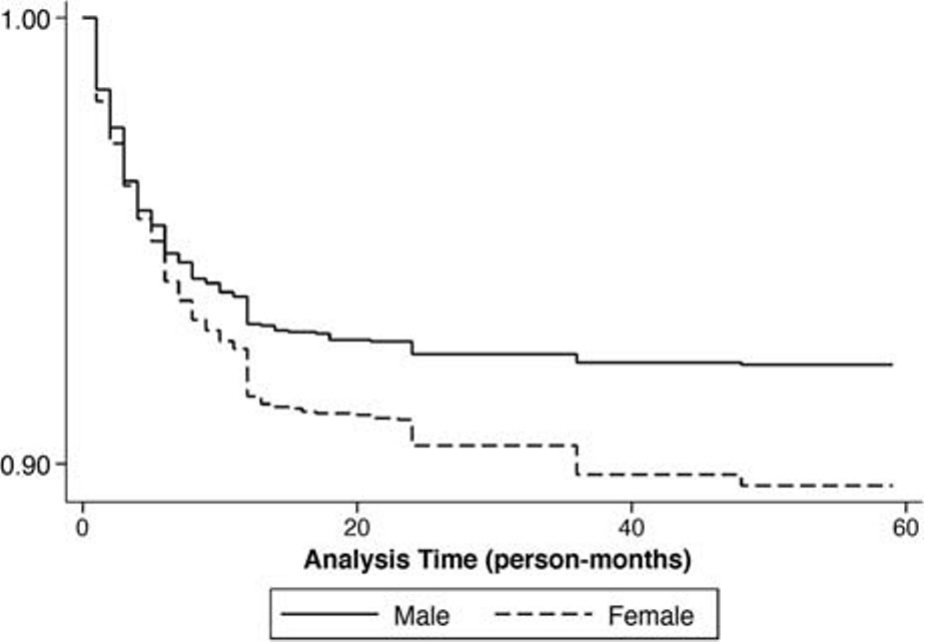
Kaplan-Meier survival estimates of time to death in person-months for male and female twins in our sample. Children enter our sample at 1 month and are censored at age at survey end or 59 months, whichever comes first. Neonatal mortality is excluded. *Source:* Created by the authors using data from the NFHS.

We disaggregate our main outcome into postneonatal infant mortality (1–11 months) and child mortality (12–59 months). Fig. 1 presents results of mixed-sex within-twin fixed-effects with postneonatal infant and child mortality as separate outcomes, showing that mortality disadvantage for females is particularly pronounced between 12 and 59 months in the first period (i.e., before 1995). Figure 2 further highlights how the survival gap between boys and girls widens after infancy. Whereas girls experience a 2 percentage point (or 23%) higher mortality probability than boys between 1 and 11 months in the first period (*p* < .10), the size of this effect for 12–59 months is 3.6 percentage points (150% higher) (*p* < .05).

### Heterogeneity in Explicit Discrimination and the Female Mortality Disadvantage by Region and Family Composition Using Within-Twin Fixed Effects

We rerun the within-twin fixed-effects models stratifying by region because we would expect to see more evidence of explicit discrimination in regions of the country where son preference has historically been the strongest, such as in northern India. Consistent with this hypothesis, we find that females experience a 3.6 percentage point (or 45%) higher probability of postneonatal under-5 mortality than males in northern India (*p* < .001) (Table 3, panel A, column 1). In contrast, the female coefficient in the pooled sample of other regions is much smaller in magnitude (slightly negative) (Table 3, panel B), suggesting stronger explicit discrimination in the north relative to other regions. These findings do not mean that there is no evidence of female mortality disadvantage in other regions of the country, given that we would actually expect a female mortality advantage in a population with no son preference (see the SSA placebo in Table 2, panel B).

Disaggregating by birth cohort, among mixed-sex twins born before 1995 (i.e., the earliest birth cohort), we find a sizable impact of explicit discrimination in the northern region, with girls experiencing a 9.1 percentage point (or 76%) higher probability of mortality than males (*p* < .001) (Table 3, panel A, column 2). In the latter two birth cohorts, the size of this mortality disadvantage attributable to explicit discrimination weakens to 0.6 and 1.9 percentage point higher probabilities of mortality compared with boys (neither coefficient is statistically significantly different from 0 at *p* < .05). Post-estimation tests of significance indicate the female coefficient in the earliest birth cohort in the northern region is significantly different from the female coefficients in the latter two birth cohorts in the northern region, which is indicative of weakening explicit discrimination over time in the region where son preference was historically strongest. However, we see a stalling of improvements in the cohorts after the mid-2000s compared with the mid-1990s in the northern region. Here, the female coefficient increases slightly between the second and third cohort, although the differences between the two cohorts are not statistically significant at *p* < .05. On the other hand, among cohorts born after the mid-2000s in the other regions, there is indication of the emergence of a female mortality advantage as the coefficient becomes negative, although the female coefficients in the other regions are not statistically significantly different by birth cohorts (Table 3, panel B). Although we see a convergence in the female coefficient for cohorts born in 1995–2005 between the northern and other regions, in the post-2005 cohorts, we see a divergence between the regions: the non-northern regions tend toward reductions in the female disadvantage, and the northern region experiences stagnation.

Next, we explore heterogeneity by family composition given literature to assess whether gender discrimination is selective by birth order and sibling composition. We rerun the within-twin fixed-effects models stratifying by number of older sisters and find that compared with males, females experience a 7.7 (96%) and 8.5 (127%) percentage point higher probability of postneonatal under-5 mortality among twins with two and three older sisters, respectively (*p* < .001) (Table 4, panel A). Post-estimation tests of significance indicate that the female coefficient in the model with no older sisters significantly differs from the female coefficients in the models with one, two, and three or more older sisters. This suggests that explicit discrimination is experienced particularly by later-born girls who have one or more sisters in the family already, rather than generally by all girls within a family. In contrast, when we conduct a placebo analysis using data from SSA, we find that there are no significant differences in the magnitude of the female coefficient across different sister combinations (Table 4, panel B).

**Table 4:** Within mixed-sex twin fixed-effects models of the association between child sex and infant and child mortality (1–59 months) disaggregated by number of older sisters for India (panel A) and SSA (panel B)

	Mortality 1–59 Months, No Older Sisters	Mortality 1–59 Months, One Older Sister	Mortality 1–59 Months, Two Older Sisters	Mortality 1–59 Months, Three Older Sisters
	(1)	(2)	(3)	(4)
A. India				
Female	–0.009	**0.025**	0.077***	0.085***
	(0.008)	**(0.013)**	**(0.019)**	**(0.024)**
Firstborn twin	–0.029***	–0.049***	–0.054**	–0.062*
	(0.008)	(0.013)	(0.019)	(0.024)
Constant	0.088***	0.110***	0.108***	0.098***
	(0.007)	(0.011)	(0.017)	(0.021)
Number of observations	3,026	1,740	930	504
*R*^2^	.009	.021	.051	.070
Number of families	1,513	870	465	252
Baseline male mortality	0.071	0.084	0.080	0.067
B. Sub-Saharan Africa				
Female	–0.026**	–0.003	–0.014	–0.024
	(0.008)	(0.009)	(0.011)	(0.012)
Firstborn twin	–0.045***	–0.043***	–0.039***	–0.031*
	(0.008)	(0.009)	(0.011)	(0.012)
Constant	0.217***	0.209***	0.201***	0.200***
	(0.007)	(0.008)	(0.010)	(0.011)
Number of observations	6,253	5,662	3,384	2,664
*R*^2^	.012	.009	.008	.007
Number of families	3,129	2,835	1,693	1,332
Baseline male mortality	0.192	0.184	0.178	0.183

*Notes:* All estimates use pooled data from the Indian National Family Health Survey (1992–1993, 1998–1999, 2005–2006, 2015–2016) and the Demographic and Health Surveys in Africa. See Table A1, online appendix, for full list of SSA countries and survey waves. The analysis was conducted in STATA 15. Bold numbers indicate a statistically significant (*p* < .05) difference between the sister category in question and no older sisters.

*p < .05; **p < .01; ***p < .001

### Supplementary Analyses

Although twin studies have widely been used to account for unobserved heterogeneity in demographic research (Guo and Tong 2006; Li et al. 2008; Marteleto and de Souza 2012; Nisén et al. 2013; Pongou 2013; Tropf and Mandemakers 2017), the external validity of estimates generated from twin data could be a concern. This might be particularly the case if the likelihood of having twins is not random, either because of genetic disposition for twins or because use of assisted reproductive technology (ART) that leads to higher rates of twinning (for dizygotic twins).^6^ To partially account for the first possibility, we exclude families with multiple twins in the family (a sign of genetic predisposition for twinning). To partially account for the second possibility, we show that the average birth year and other demographic characteristics for both mixed-sex and same-sex twins are very similar (see online appendix Table A4, panels B and C for same-sex twins). This finding suggests that ART is not driving the composition of mixed-sex twins, which would affect the mixed-sex twins’ sample (entirely dizygotic) more than the same-sex sample (both monozygotic and dizygotic).^7^ Although ART has increased over time in India, it is still concentrated to a relatively small urban elite, and thus it is highly unlikely that those who practice ART would be a large enough group to change the trend for the whole country over the long period of consideration (Smits and Monden 2011). Our samples show consistent known maternal factors associated with spontaneous twinning of maternal age and birth order (Hoekstra et al. 2008). And in our sample, both same- and mixed-sex twins have mothers who are older and, likely in relation to this age pattern, have somewhat higher education than singletons. Perhaps the most striking difference between twin births and singleton births is the prevalence of infant and child mortality—a finding that holds for both same- and mixed-sex twins. This corresponds with literature suggesting that twins might be more vulnerable to mortality in infancy or childhood (Monden and Smits 2017).

As Table 1 shows, mixed-sex twins are increasingly born into different types of households (more educated, smaller sibship size, etc.) over time, but Table A4 in the online appendix shows that this is true for singletons and same-sex twins as well, thus indicating that the household characteristics of *both* singletons and twins are changing. This is further confirmed in Table A5 in the online appendix, which shows that the interaction terms between family characteristics and birth cohort in predicting mixed-sex twin births are not changing significantly differently for twins than for singletons. The sole exception is the significant positive interaction between the last birth cohort and mother’s tertiary education. This positive interaction appears only when the last three years (2014–2016) of births in our sample are included. Reestimating the main models shown in Table 2 with these years excluded yields substantively similar results (see Table A7, online appendix).

We also conduct an ordinary least squares (OLS) regression analysis regressing mortality on child sex for a large sample of singleton children and using within-family fixed-effects (panels A and B of Table A6, online appendix). In the OLS and within-family fixed-effects models, we find that females experience a 0.7 and 0.6 percentage point (about 20%) higher probability of postneonatal under-5 mortality than males (*p* < .001), net of controls for family and child characteristics, respectively. Although we are hesitant to directly compare these OLS results with those generated by the within-twin fixed effects, we cautiously note that the female disadvantage in mortality, both in absolute and relative measures, indicated by the magnitude of the coefficient on the pooled OLS models is smaller than those on the within-twin fixed-effects models. This difference is especially so for cohorts born prior to 1995. Although they used a different estimation strategy than ours to exploit random variation in the sex of the child, Barcellos et al. (2014) also found that estimates of gender discrimination in health investments and breastfeeding indicated in their experimental sample is higher than in standard OLS estimates before 1992. This suggests that in this period, OLS estimates likely overstate the role of implicit discrimination processes when estimating the female mortality disadvantage given that completed family size and birth order variables are likely to capture some of the excluded explicit discrimination processes as well. Interestingly, the opposite pattern is visible for the cohorts born in 1995–2005, for which the relative female disadvantage in mortality indicated by the within-twin fixed-effects model is smaller than that OLS estimates, suggesting that actually explicit discrimination is weaker than OLS estimates would suggest.

In the main analysis, we do not include controls for birth weight because of the very high missing values for this variable (e.g., missing for more than 80% of respondents in the pooled sample), but our indicator of firstborn twin likely captures whether the twin had higher or lower birth weight because of the correlation between twin birth order and birth weight. We nevertheless perform sensitivity tests with imputed birth weight and rerun our analyses; results are unchanged (panel E of Table A2, online appendix).

### Limitations

One limitation of the within-twin fixed-effects approach is the potential for unobserved confounders that vary across mixed-sex twins. Unlike identical (monozygotic) twins, who share 100% of their genetic material, mixed-sex twins are fraternal (dizygotic) and share 50% of their genetic material (about the same amount of genetic material that nontwin siblings share). Thus, it is plausible that there are unobservable genetic differences between twins that lead to differential parental care and attention, net of child sex. Another biological mechanism that may operate distinctively among mixed-sex twins is intrauterine hormonal exposure (Ahrenfeldt et al. 2019; Tapp et al. 2011). According to this mechanism, girls in mixed-sex twins may experience high levels of prenatal testosterone exposure and consequently may be more masculinized in their development than singleton or same-sex twin girls. Conversely, boys exposed to a female co-twin may be more feminized because of estrogen exposure. Evidence for this hypothesis in relation to early-life mortality outcomes is limited and, when available, is mixed and inconclusive (Ahrenfeldt et al. 2017; Pongou 2013). Nevertheless, throughout our analysis, by comparing mixed-sex twins with each other and conducting our placebo analysis also on SSA mixed-sex twins, we obtain results that are unaffected even if sex differences in mixed-sex twin populations may be less pronounced than in singleton populations.

It is plausible that twins may be a greater negative shock than singleton births, leading twins to receive differential treatment than other types of children. If so, we could interpret our estimates to be upper-bound estimates of explicit discrimination, given that, as described before, twins do not differ greatly from other children on most observable characteristics. Even if overall mortality outcomes are worse for twins, we should not expect a female disadvantage in mortality among females in mixed-sex twins in the absence of son preference. Ultimately, the exceptional nature of twin births is what makes them so interesting for our experimental design by allowing us to control for differential family selectivity into having a less desired female child.

A final limitation is that our analysis provides a useful way to measure the mortality impact of explicit discrimination net of implicit discrimination, but it does not shed much insight into the specific mechanisms through which explicit discrimination operates. Although we hypothesize that differential allocation of resources is the main mechanism through which the explicit discrimination that leads to elevated female mortality among mixed-sex twins operates, it is difficult to test this directly using NFHS data and the within-twin fixed-effects approach because of significant data limitations. The NFHS collects early childhood health and nutrition measures for children born only in the last five years, leaving us with a small sample of twins with full nutrition and health information. More generally, it is plausible that the accumulated differences in resource investment that result in higher female mortality take place over a much longer time span (e.g., months or years) and occur in ways that are not easily captured in survey instruments such as the NFHS. This may also help explain why female mortality disadvantage in studies is more consistently found whereas evidence for differential allocation is more mixed. Parents might also be prone to reporting bias when describing allocation of resources, particularly for children born several years prior to the survey, and may also be reluctant to report differential investments in children. Even for children born in the last five years, the NFHS data do not have information on key measures (e.g., height for age) for deceased children. Furthermore, children who have died will likely differ in key characteristics (e.g., they might have been breastfed less or have a lower probability of vaccination) than children who survived through early childhood, and it would not be possible to know whether these differences led to death (e.g., they died because they were not immunized) or instead early death led to these differences (e.g., they would have been immunized if they had survived longer). Finally, if a child died, the surviving twin may have received better treatment because of the death (e.g., parents may dote on the offspring who survives to compensate for the loss ex post facto). Ultimately, because mortality is the strongest benchmark of discrimination—one that is least prone to recall bias—and it is what our data best allow us to measure, it is the focus of our research presented here.

## Discussion

One of the most striking demographic manifestations of son preference in India is the persistence of excess female under-5 mortality. Although considerable literature has attributed the under-5 female mortality disadvantage to parents differentially investing more resources in boys versus girls within families—*explicit discrimination*—this literature has not adequately controlled for what we term *implicit discrimination* processes that sort girls into different types of families (e.g., larger, poorer, or with varying son preference) and at different birth orders than boys. It is conceptually important to recognize these two distinctive micro-level mechanisms of discrimination because the family-level processes implied by each are different, as are the policy responses they require. To better address the endogeneity associated with implicit discrimination processes, we explore the association between child sex and postneonatal under-5 mortality using a sample of mixed-sex twins. We argue that mixed-sex twins provide a natural experiment that exogenously assigned a boy and a girl to families at the same time, thus controlling for family selectivity into having an unwanted female child, birth order, and other implicit discrimination processes.

Our within-twin fixed-effects models show that female children experienced a significantly higher probability of postneonatal under-5 mortality. This provides strong evidence of the important role of explicit discrimination in the female mortality disadvantage observed in our data because our models control for implicit discrimination processes that resulted in the differential sorting of boys and girls into different kinds of families and birth orders. The Indian estimates for explicit discrimination are particularly striking when compared with a placebo analysis conducted in sub-Saharan Africa, where female twins actually had a survival advantage; this finding corresponds with literature showing that males have a biological disadvantage in early life.

Using our novel measure, we find that that the role of explicit discrimination underlying the female mortality disadvantage weakened for cohorts born after the mid-1990s relative to those born prior to mid-1990s. Subsequent analyses also show that our temporal results are largely driven by northern India, and explicit discrimination declined over time in this region, which has historically been characterized by high son preference. Nevertheless, our results do not indicate the disappearance of a mortality disadvantage attributable to explicit discrimination in India, and indeed the cohorts born after the mid-2000s in northern India appear to show stalling improvements.

Although we are not able to test them directly, we anticipate that a combination of contextual changes—weakening son preference, policy initiatives aimed at improving girls’ status, fertility decline, and the ability to realize son preference at lower parities due to the practice of sex-selective abortion—underpin weakening explicit discrimination in India since the mid-1990s. Compared with the pre-1995 period, we find that later cohorts of mixed-sex twins are born into smaller families with lower indicators of stated son preference. Although these results refer to stated preference indicators, it is plausible that son preference has weakened through socioeconomic development (Chung and Das Gupta 2007; Kashyap and Villavicencio 2016) via channels such as improved educational and economic opportunities for women (Bhat and Zavier 2003; Luke and Munshi 2011; Murthi et al. 1995; Pande and Astone 2007) and media exposure (Jensen and Oster 2009; Lin and Adserà 2013; Ting et al. 2014). Furthermore, since the 1990s, several states across India have launched financial incentive policies to encourage investments in daughters’ health and education. Implementation of these policies has been irregular, and their systematic review has been limited (Sekher 2012), but available evidence suggests improvements in postnatal outcomes after their implementation (Sekher and Ram 2015; Sinha and Yoong 2009; Srinivasan and Bedi 2009).

Research has found that weakening son preference is correlated with fertility decline (Bhat and Zavier 2003). With reductions in overall family size, differences in resource allocations may become less pronounced, particularly given our finding that it is girls at higher birth orders with existing sisters who are most vulnerable to explicit discrimination. In part, explicit discrimination also could have weakened not because son preference weakened but rather because parents were able to realize son preference with their desire for smaller families through sex-selective abortion (e.g., Anukriti et al. 2018; Kashyap 2019). In a context with strong son preference, such as India, fertility decline may be enabled by sex-selective abortion. Although the mechanism of opting out of having unwanted daughters does not apply to mixed-sex twins in the same way as it does to other births, which precisely makes our strategy better at controlling for this form of implicit discrimination, parents with mixed-sex twins could opt out of having other children and achieve their preferred sex ratio with fewer overall children.

The preceding discussion suggests that targeting son preference solely through policies that ban prenatal sex selection may be counterproductive for reducing explicit discrimination. The uneven adoption of sex-selective abortion among more advantaged households, however, could imply that girls are increasingly selected into the most disadvantaged households, which may underpin continued explicit discrimination. The fact that improvements appear to have stalled among more recent cohorts since the mid-2000s suggests that policy responses that target health investments in girls specifically (such as through financial incentive schemes), rather than generalized poverty policies targeting all children, are still necessary. These policies, combined with indirect measures to weaken son preference through media advocacy as well as measures to improve women’s outcomes in political, legal, and economic domains are needed to further reduce explicit discrimination.

Although the approach presented here does not provide an all-encompassing measure of son preference (indeed, it is one of several ways to explore son preference), we provide a conceptual contribution by distinguishing between explicit and implicit discrimination processes, and we demonstrate a quasi-experimental approach to better estimate explicit discrimination effects using a novel sample. It is fundamental for our understanding of son preference to explore whether the effects of son preference arise from parents’ actively investing more in their boys than their girls or instead they accrue as a result of changing patterns of implicit discrimination. A significant contribution of our approach is that it allows for the temporal analysis of the impact of explicit discrimination on the female mortality disadvantage in India over four decades.

Although our analysis is focused on India, the points we raise about the different processes behind both explicit and implicit discrimination can also be applied to other contexts in South, East, and Central Asia with high son preference. This distinction may become particularly important as wider changes such as fertility decline and technology diffusion continue across countries with historically strong son preference, implying that changing family selectivity—as opposed to differential resource allocation within families—could become a particularly important mechanism through which the mortality manifestations of son preference emerge. Future research should explore the mechanisms of both explicit and implicit discrimination and better understand the interrelationship between changing son preference, fertility decline, and excess female mortality.

## Electronic supplementary material

ESM 1 (PDF 478 kb)

## Data Availability

The data sets used in the study are publicly available to registered users on the website of the Demographic and Health Surveys (DHS) program (https://dhsprogram.com/data/available-datasets.cfm).

## Footnotes

1 The male/female ratio (hereafter, *sex ratio*) of infant mortality is generally higher than 1, indicative of higher mortality for males than for females. The same pattern also exists in early childhood (between ages 1 and 4 years), although sex ratios are less masculine in most populations in this age group compared with infancy (first 12 months of life). In the first year of life, newborn girls have a biological advantage over boys because of their lower vulnerability to perinatal conditions, congenital abnormalities, and certain infectious diseases, such as intestinal and lower respiratory infections (Drevenstedt et al. 2008).

2 Throughout the article, to be consistent with demographic convention (e.g., sex ratios, sex-specific mortality) and the variable in the National Family Health Survey (NFHS), we use sex of the child. We recognize nevertheless that the female mortality disadvantage is the product of social—rather than biological—processes related to *gender*-based discrimination.

3 Sex selection at first birth in northern India has been noted in recent work (e.g., Anukriti 2018) that challenges this assumption.

4 Unlike Anukriti et al. (2018), we also use the most recent wave of the NFHS-4, and our sample covers more recent births as well (i.e., after 2005).

5 The states included in the northern category include Bihar, Delhi, Gujarat, Haryana, Himachal Pradesh, Jammu and Kashmir, Madhya Pradesh, Maharashtra, Punjab, Rajasthan, and Uttar Pradesh.

6 ART has predominantly been associated with increased rates of dizygotic twinning. Dizygotic twins may be mixed sex or the same sex, but all monozygotic twins are the same sex (Pison et al. 2015).

7 We are unable to measure zygosity in the NFHS data.
