# Supplementary material for: Gender Discrimination and Excess Female Under-5 Mortality in India: A New Perspective Using Mixed-Sex Twins
Source: Demography. 2020 Sep 25;57(6):2143–67. doi: 10.1007/s13524-020-00909-0 (PMC7732804; doi:10.1007/s13524-020-00909-0)
Supplement: Supplementary file 1 — (PDF 478 kb) [file 13524_2020_909_MOESM1_ESM.pdf]

Online Appendix

Gender Discrimination and Excess Female Under-5 Mortality in India: A New Perspective Using Mixed-Sex Twins

Ridhi Kashyap and Julia Behrman

Table A1. Overview of Sub-Saharan Africa DHS survey year and samples used

**Country**	**Year**	**Year**	**Year**	**Year**	**Year**	**Year**
Benin	1996	2001	2006	2011-12		
Burkina Faso	1993	1998-99	2003	2010		
Cameroon	1991	1998	2004	2011		
Ghana	1993	1998	2003	2008	2014	
Kenya	1993	1998-99	2003	2008	2014	
Madagascar	1992	1997	2003-04	2008-09		
Malawi	1992	2000	2004	2010		
Mali	1995-96	2001	2006	2012-13		
Namibia	1992	2000	2006-07	2013		
Niger	1992	1998	2006	2012		
Nigeria	1990	2003	2008	2013		
Rwanda	1992	2000	2005	2010	2014-15	
Senegal	1992	1997	2005	2010-11	2012-13	
Tanzania	1991-92	1996	1999	2004-05	2010	
Uganda	1995	2000-01	2006	2011		
Zambia	1992	1996	2001-02	2006	2013-14	
Zimbabwe	1994	1999	2005-06	2010-11		

Table A2. Alternative specifications of within mixed sex twin fixed effects models of the association between child sex and infant and child mortality (1-59 months) in India; Panel A uses logistic regression fixed effects with results presented as odds ratios; Panel B uses Cox proportional hazard models with fixed effects with results presented as hazard ratios; Panel C uses linear regression fixed effects, but limits to births in the 10 years before the survey; Panel D uses linear regression fixed effects, but uses an alternative specification of birth cohorts; Panel E uses linear regression fixed effects, with a control for birth weight created through multiple imputation. Estimates use pooled data from the Indian National Family Health Survey (1992-1993, 1998-1998, 2005-2006, 2015-2016). Analysis conducted in STATA 15.

Panel A. Logistic regression fixed effects	(1)	(2)	(3)	(4)
	Mortality 1-59 mos.	Mortality 1-59 mos.	Mortality 1-59 mos.	Mortality 1-59 mos.
	Pooled	Born before 1995	Born 1995-2005	Born after 2005
Female	1.468***	1.891***	1.088	0.980
	(0.160)	(0.301)	(0.219)	(0.263)
First born twin	0.514***	0.680*	0.397***	0.311***
	(0.056)	(0.108)	(0.080)	(0.083)
Observations	782	366	248	168
Number of families	391	183	124	84

Panel B. Cox proportional hazards	(1)	(2)	(3)	(4)
	Age at death (months)	Age at death (months)	Age at death (months)	Age at death (months)
	Pooled	Born before 1995	Born 1995-2005	Born after 2005
Female	1.427***	2.006***	1.094	0.685
	(0.146)	(0.290)	(0.207)	(0.181)
First born twin	0.420***	0.716*	0.280***	0.146***
	(0.044)	(0.103)	(0.056)	(0.042)
Observations	6,190	1,866	2,278	2,046
Number of families	3095	933	1139	1023

Panel C. Limiting to births in last 10 years	(1)	(2)	(3)	(4)
	Mortality 1-59 mos.	Mortality 1-59 mos.	Mortality 1-59 mos.	Mortality 1-59 mos.
	Pooled	Born before 1995	Born 1995-2005	Born after 2005
Female	0.022*	0.102***	**-0.006**	**0.007**
	(0.009)	(0.027)	**(0.022)**	**(0.009)**
First born twin	-0.019*	0.043	-0.001	-0.042***
	(0.009)	(0.027)	(0.022)	(0.009)

Constant	0.077***	0.076**	0.100***	0.069***
	(0.008)	(0.025)	(0.020)	(0.008)
Observations	3,298	600	644	2,054
R-squared	0.007	0.049	0.000	0.023
Number of families	1,649	300	322	1,027
Baseline male mortality	0.066	0.100	0.099	0.046

Panel D. Alternative birth cohorts	(1)	(2)	(3)	(4)
	Mortality 1-59 mos.	Mortality 1-59 mos.	Mortality 1-59 mos.	Mortality 1-59 mos.
	Pooled	Born before or in 1980s	Born 1990s	Born in 2000s
Female	0.020**	0.063**	**0.014**	**0.009**
	(0.006)	(0.020)	**(0.013)**	**(0.007)**
First born twin	-0.039***	-0.014	-0.047***	-0.042***
	(0.006)	(0.020)	(0.013)	(0.007)
Constant	0.097***	0.145***	0.112***	0.073***
	(0.006)	(0.018)	(0.012)	(0.006)
Observations	6,200	1,142	1,676	3,382
R-squared	0.017	0.020	0.019	0.022
Number of families	3,100	571	838	1,691
Baseline male mortality	0.075	0.137	0.086	0.050

Panel E. Including birth weight	(1)	(2)	(3)	(4)
	Mortality 1-59 mos.	Mortality 1-59 mos.	Mortality 1-59 mos.	Mortality 1-59 mos.
	Pooled	Born before 1995	Born 1995-2005	Born after 2005
Female	0.020**	0.053***	**0.006**	**0.007**
	(0.006)	(0.015)	**(0.010)**	**(0.009)**
First born twin	-0.039***	-0.022	-0.047***	-0.042***
	(0.006)	(0.015)	(0.010)	(0.009)
Birth weight	-0.000	-0.000	-0.000	-0.000
	(0.000)	(0.000)	(0.000)	(0.000)
Constant	0.103***	0.131**	0.106***	0.076*
	(0.021)	(0.048)	(0.032)	(0.032)
Observations	6,200	1,868	2,278	2,054
Number of families	3,100	934	1,139	1,027
Baseline male mortality	0.075	0.118	0.068	0.046

*** p<0.001, ** p<0.01, * p<0.05

Notes: Bold numbers indicate statistically significant ($p<0.05$) difference between the birth cohort in question and the first birth cohort (e.g. born before 1995). In Panel B, respondents are censored at age at survey or 59 months (whichever comes first).

Table A3. Additional information on sub-Saharan African sample; Panel A presents descriptive summary of background characteristics of mixed-sex twins for sub-Saharan Africa sample, including tests for significant difference between the first birth cohort and the two subsequent cohorts (estimates use sampling weights provided by the DHS); Panel B presents difference-in-difference analysis using pooled Africa and India data and an interaction between female and India.

Panel A.	(1)<br>*Pooled*	(2)<br>*Born before 1995*	(3)<br>*Born 1995-2005*	(4)<br>*Born after 2005*
Mortality (1-59 mos.)	0.18	0.24	**0.17**	**0.09**
Female	0.50	0.50	0.50	0.50
Birth year	1996	1987	**2000**	**2009**
Birth order	4.46	4.39	**4.53**	4.45
Rural	0.72	0.74	0.73	**0.67**
Mother no school	0.47	0.51	**0.46**	**0.42**
Mother primary school	0.36	0.37	0.36	**0.33**
Mother secondary school	0.15	0.11	**0.16**	**0.21**
Mother tertiary	0.02	0.01	**0.02**	**0.04**
Mother age at birth	27.66	26.36	**28.17**	**29.23**
Total children born to mother	6.52	7.39	**6.26**	**5.27**
Mother's ideal number of boys	2.49	2.59	**2.50**	**2.32**
Mother's ideal number of girls	2.34	2.47	**2.35**	**2.12**
Mother's ideal sex ratio	0.51	0.51	**0.52**	**0.52**
N	17,963	7,124	7,533	3,306

Notes: All measures are dichotomous except birth year (ranges from 1960 to 2014), birth order (ranges from 1 to 14), mother age at birth (ranges from 10 to 47), total children born (ranges from 1 to 16), mother's ideal boys (ranges from 0 to 20), mother's ideal girls (ranges from 0 to 15), and ideal sex ratio (ranges from 0 to 1 and excludes women who desire zero children). Bold numbers indicate statistically significant (p<0.05) difference between the birth cohort in question and the first birth cohort (e.g. born before 1995). Two-sample t tests for all continuous outcomes, and chi-square tests for all dichotomous outcomes. Singleton sample all live in households with no other twins.

Panel B.	(1)<br>Mortality<br>1-59 mos.
Female	-0.016**
	(0.005)
First born twin	-0.040***
	(0.005)
India	-0.094***
	(0.008)
Female*India	0.035***
	(0.011)
Born 1995-2005 (ref=before 1995)	-0.071***
	(0.005)
Born after 2005 (ref=before 1995)	-0.130***
	(0.006)
Constant	0.264***
	(0.005)

Observations	24,163
R-squared	0.033
Baseline level of male mortality	0.157

*** p<0.001, ** p<0.01, * p<0.05

Table A4. Descriptive summary of background characteristics of singletons (Panel A); female same-sex twins (Panel B); and male same-sex twins (Panel C) including tests for significant difference between the first birth cohort and the two subsequent cohorts. All estimates use pooled data from the Indian National Family Health Survey (1992-1993, 1998-1998, 2005-2006, 2015-2016) and use sampling weights provided by the NFHS.

Panel A.	Singleton sample:			
	(1)	(2)	(3)	(4)
	Pooled	*Born before 1995*	*Born 1995-2005*	*Born after 2005*
Mortality (1-59 mos.)	0.04	0.07	**0.03**	**0.02**
Female	0.48	0.48	0.48	0.48
Birth year	1996	1985	**2000**	**2010**
Birth order	2.43	2.48	2.48	**2.29**
Rural	0.72	0.72	**0.71**	**0.71**
Northern region	0.60	0.59	**0.60**	**0.60**
Hindu	0.80	0.81	**0.79**	**0.78**
Poorest 40%	0.44	0.40	**0.46**	**0.48**
Mother no school	0.52	0.63	**0.51**	**0.35**
Mother primary school	0.16	0.16	**0.15**	**0.15**
Mother secondary school	0.27	0.18	**0.29**	**0.42**
Mother tertiary	0.05	0.02	**0.05**	**0.09**
Mother age at birth	23.29	22.14	**23.75**	**24.61**
Total children born to mother	3.83	4.49	**3.70**	**2.86**
Mother's ideal number of boys	1.40	1.54	**1.35**	**1.24**
Mother's ideal number of girls	1.06	1.10	**1.04**	**1.00**
Mother's ideal sex ratio	0.57	0.58	**0.56**	**0.55**
N	2004684	803490	692572	508622

Notes: All measures are dichotomous except birth year (ranges from 1954 to 2016), birth order (ranges from 1 to 18), mother age at birth (ranges from 10 to 50), total children born (ranges from 1 to 18), mother's ideal boys (ranges from 0 to 20), mother's ideal girls (ranges from 0 to 11), and ideal sex ratio (ranges from 0 to 1 and excludes women who desire zero children). Bold numbers indicate statistically significant (p<0.05) difference between the birth cohort in question and the first birth cohort (e.g. born before 1995). Two-sample t tests for all continuous outcomes, and chi-square tests for all dichotomous outcomes.

Panel B.	Female same sex twin sample:			
	(1)	(2)	(3)	(4)
	Pooled	*Born before 1995*	*Born 1995-2005*	*Born after 2005*
Mortality (1-59 mos.)	0.12	0.20	**0.09**	**0.06**
Female	1.00	1.00	1.00	1.00
Birth year	1999	1986	**2000**	**2011**
Birth order	3.04	3.27	**3.03**	**2.82**
Rural	0.66	0.68	0.64	0.67
Northern region	0.55	0.51	**0.55**	**0.60**

Hindu	0.76	0.77	0.77	0.74
Poorest 40%	0.40	0.33	**0.38**	**0.49**
Mother no school	0.45	0.57	**0.41**	**0.36**
Mother primary school	0.15	0.16	0.18	**0.12**
Mother secondary school	0.33	0.23	**0.35**	**0.40**
Mother tertiary	0.07	0.04	0.06	**0.12**
Mother age at birth	24.43	23.26	**24.65**	**25.36**
Total children born to mother	4.48	5.15	**4.53**	**3.74**
Mother's ideal number of boys	1.34	1.46	**1.31**	**1.27**
Mother's ideal number of girls	1.14	1.18	1.18	**1.06**
Mother's ideal sex ratio	0.54	0.55	**0.53**	0.54
N	6149	1991	2134	2024

Notes: All measures are dichotomous except birth year (ranges from 1962 to 2016), birth order (ranges from 1 to 13), mother age at birth (ranges from 12 to 48), total children born (ranges from 2 to 14), mother's ideal boys (ranges from 0 to 8), mother's ideal girls (ranges from 0 to 6), and ideal sex ratio (ranges from 0 to 1 and excludes women who desire zero children). Bold numbers indicate statistically significant (p<0.05) difference between the birth cohort in question and the first birth cohort (e.g. born before 1995). Two-sample t tests for all continuous outcomes, and chi-square tests for all dichotomous outcomes.

Panel C.	Male same sex twin sample:			
	(1)	(2)	(3)	(4)
	Pooled	*Born before 1995*	*Born 1995-2005*	*Born after 2005*
Mortality (1-59 mos.)	0.09	0.17	**0.07**	**0.05**
Female	0.00	0.00	0.00	0.00
Birth year	1999	1986	**2000**	**2011**
Birth order	3.19	3.39	3.29	**2.86**
Rural	0.67	0.66	0.67	0.69
Northern region	0.54	0.53	0.55	0.53
Hindu	0.77	0.81	0.78	**0.73**
Poorest 40%	0.36	0.33	**0.39**	0.37
Mother no school	0.45	0.58	**0.48**	**0.27**
Mother primary school	0.14	0.16	0.13	**0.13**
Mother secondary school	0.33	0.19	**0.31**	**0.49**
Mother tertiary	0.08	0.06	0.07	**0.11**
Mother age at birth	24.95	23.69	**25.34**	**25.79**
Total children born to mother	4.33	5.09	**4.31**	**3.56**
Mother's ideal number of boys	1.42	1.54	**1.44**	**1.29**
Mother's ideal number of girls	1.02	1.04	1.03	**0.97**
Mother's ideal sex ratio	0.58	0.59	0.58	**0.57**
N	6469	2074	2310	2085

Notes: All measures are dichotomous except birth year (ranges from 1961 to 2016), birth order (ranges from 1 to 13), mother age at birth (ranges from 10 to 44), total children born (ranges from 2 to 15), mother's ideal boys (ranges from 0 to 9), mother's ideal girls (ranges from 0 to 8), and ideal sex ratio (ranges from 0 to 1 and excludes women who desire zero children). Bold numbers indicate statistically significant ($p<0.05$) difference between the birth cohort in question and the first birth cohort (e.g. born before 1995). Two-sample t tests for all continuous outcomes, and chi-square tests for all dichotomous outcomes.

Table A5. Analysis of the factors predicting having a mixed sex twin birth (compared to singleton birth) using linear probability models and including interactions between family characteristics and birth period. All estimates use pooled data from the Indian National Family Health Survey (1992-1993, 1998-1998, 2005-2006, 2015-2016) and use sampling weights provided by the NFHS.

	(1)	(2)	(3)	(4)	(5)	(6)	(7)
	Mixed-sex twin birth	Mixed-sex twin birth	Mixed-sex twin birth	Mixed-sex twin birth	Mixed-sex twin birth	Mixed-sex twin birth	Mixed-sex twin birth
Born 1995-2005 (ref=before 1995)	0.001***	0.001**	0.001*	0.000	0.001*	0.001**	0.000
	(0.000)	(0.000)	(0.000)	(0.000)	(0.000)	(0.000)	(0.000)
Born after 2005 (ref=before 1995)	0.002***	0.001	0.001	0.000	0.001	0.000	0.001
	(0.000)	(0.000)	(0.001)	(0.001)	(0.000)	(0.000)	(0.000)
Rural		0.000	0.000	0.000	0.000	0.000	0.000
		(0.000)	(0.000)	(0.000)	(0.000)	(0.000)	(0.000)
Hindu		-0.000	-0.000	-0.000	-0.000	-0.000	-0.000
		(0.000)	(0.000)	(0.000)	(0.000)	(0.000)	(0.000)
Northern India		0.000	0.000	0.000	0.000	0.000	0.001
		(0.000)	(0.000)	(0.000)	(0.000)	(0.000)	(0.000)
Poorest 40%		0.000	0.000	0.000	0.000	0.000	0.000
		(0.000)	(0.000)	(0.000)	(0.000)	(0.000)	(0.000)
Mother primary (ref=no school)		0.002***	0.002***	0.002***	0.002***	0.002***	0.001
		(0.001)	(0.001)	(0.001)	(0.001)	(0.001)	(0.001)
Mother secondary (ref=no school)		-0.000*	-0.000*	-0.000*	-0.000*	-0.000	-0.000
		(0.000)	(0.000)	(0.000)	(0.000)	(0.000)	(0.000)
Mother tertiary (ref=no school)		-0.000	-0.000	-0.000	-0.000	-0.000	-0.000
		(0.000)	(0.000)	(0.000)	(0.000)	(0.000)	(0.000)
Born 1995-2005*Rural			-0.000				
			(0.000)				
Born after 2005*Rural			-0.000				
			(0.001)				
Born 1995-2005*Hindu				0.000			
				(0.000)			
Born after 2005*Hindu				0.001			
				(0.001)			
Born 1995-2005*North					-0.000		
					(0.000)		
Born after 2005*North					0.000		
					(0.000)		
Born 1995-2005*Poorest						-0.001	
						(0.000)	
Born after 2005*Poorest						0.000	
						(0.000)	
Born 1995-2005*Mother primary							0.000
							(0.001)
Born after 2005*Mother primary							0.000
							(0.000)
Born 1995-2005*Mother secondary							0.001

							(0.001)
Born after 2005*Mother secondary							-0.001
							(0.001)
Born 1995-2005*Mother tertiary							-0.000
							(0.000)
Born after 2005*Mother tertiary							0.003*
							(0.001)
Observations	2,010,880	2,010,880	2,010,880	2,010,880	2,010,880	2,010,880	2,010,880
R-squared	0.000	0.000	0.000	0.000	0.000	0.000	0.000

Robust standard errors in parentheses clustered at the family level

*** p<0.001, ** p<0.01, * p<0.05

Notes: Models 2-7 include controls for birth year, female, and survey round (not shown). 4 observations from the twin sample are dropped due to missing information on background covariates.

Table A6. OLS regression models of the association between child sex and infant and child mortality (1-59 months) using a sample of singleton children (Panel A); within family fixed effects estimates of the association between child sex and infant and child mortality (1-59 months) (Panel B). All estimates use pooled data from the Indian National Family Health Survey (1992-1993, 1998-1998, 2005-2006, 2015-2016). Analysis conducted in STATA 15.

Panel A. OLS	(1)	(2)	(3)	(4)
	Mortality 1-59 mos.	Mortality 1-59 mos.	Mortality 1-59 mos.	Mortality 1-59 mos.
	Pooled	Born before 1995	Born 1995-2005	Born after 2005
Female	0.007***	0.011***	**0.005*****	**0.002*****
	(0.000)	(0.001)	**(0.000)**	**(0.000)**
Background controls	YES	YES	YES	YES
Observations	2,004,684	803,490	692,572	508,622
R-squared	0.021	0.023	0.008	0.005
Baseline male mortality	0.036	0.056	0.027	0.019

*** p<0.001, ** p<0.01, * p<0.05

Notes: Background controls include rural, northern region, mother's school, poorest 40%, birth year, birth order, and survey round. Bold numbers indicate statistically significant (p<0.05) difference between the birth cohort in question and the first birth cohort (e.g. born before 1995). Robust standard errors clustered at the family level. Neonatal mortality excluded to correspond with within twin FE estimates.

Panel B. Within family FE	(1)	(2)	(3)	(4)
	Mortality 1-59 mos.	Mortality 1-59 mos.	Mortality 1-59 mos.	Mortality 1-59 mos.
	Pooled	Born before 1995	Born 1995-2005	Born after 2005
Female	0.006***	0.012***	**0.005*****	**0.001**
	(0.000)	(0.001)	**(0.001)**	**(0.001)**
Background controls	YES	YES	YES	YES
Observations	1,971,714	787,717	681,111	502,886
R-squared	0.007	0.008	0.002	0.004
Number of families	704,348	293,432	343,664	289,148
Baseline male mortality	0.036	0.065	0.034	0.021

*** p<0.001, ** p<0.01, * p<0.05

Notes: Background controls include birth order and year of birth. Bold numbers indicate statistically significant (p<0.05) difference between the birth cohort in question and the first birth cohort (e.g. born before 1995). Excludes families with twins. Neonatal mortality excluded to correspond with within twin FE estimates.

Table A7. Within mixed sex twin fixed effects models of the association between child sex and infant and child mortality (1-59 months) in India, excluding twins born in 2014-2016. Estimates use pooled data from the Indian National Family Health Survey (1992-1993, 1998-1998, 2005-2006, 2015-2016). Analysis conducted in STATA 15.

	(1)	(2)	(3)	(4)
	Mortality 1-59 mos.	Mortality 1-59 mos.	Mortality 1-59 mos.	Mortality 1-59 mos.
	Pooled	Born before 1995	Born 1995-2005	Born after 2005
Female	0.022***	0.053***	0.006	0.011
	(0.007)	(0.015)	(0.010)	(0.010)
First born twin	-0.039***	-0.023	-0.047***	-0.046***
	(0.007)	(0.015)	(0.010)	(0.010)
Constant	0.100***	0.131***	0.093***	0.071***
	(0.006)	(0.013)	(0.009)	(0.009)
Observations	5,800	1,868	2,278	1,654
R-squared	0.017	0.019	0.021	0.027
Number of families	2,900	934	1,139	827
Baseline male mortality	0.078	0.118	0.068	0.046

*** p<0.001, ** p<0.01, * p<0.05

Notes: Bold numbers indicate statistically significant (p<0.05) difference between the birth cohort in question and the first birth cohort (e.g. born before 1995).
